# Identification of biomarkers and pathways in hypertensive nephropathy based on the ceRNA regulatory network

**DOI:** 10.1186/s12882-020-02142-8

**Published:** 2020-11-11

**Authors:** Zhen Wang, Zhongjie Liu, Yingxia Yang, Lei Kang

**Affiliations:** 1 Nephrology Department, Dongzhimen Hospital Affiliated to Beijing University of Chinese Medicine, No.5 Haiyuncang Road, Dongcheng District, Beijing, 100700 China; 2 Neurology Department, Dongzhimen Hospital Affiliated to Beijing University of Chinese Medicine, No.5 Haiyuncang Road, Dongcheng District, Beijing, 100700 China

**Keywords:** Hypertensive nephropathy, Differentially expressed RNAs, ceRNA network directly associated with HTN, Functional analysis

## Abstract

**Background:**

Hypertensive nephropathy (HTN) is a kind of renal injury caused by chronic hypertension, which seriously affect people’s life. The purpose of this study was to identify the potential biomarkers of HTN and understand its possible mechanisms.

**Methods:**

The dataset numbered GSE28260 related to hypertensive and normotensive was downloaded from NCBI Gene Expression Omnibus. Then, the differentially expressed RNAs (DERs) were screened using R limma package, and functional analyses of DE-mRNA were performed by DAVID. Afterwards, a ceRNA network was established and KEGG pathway was analyzed based on the Gene Set Enrichment Analysis (GSEA) database. Finally, a ceRNA regulatory network directly associated with HTN was proposed.

**Results:**

A total of 947 DERs were identified, including 900 DE-mRNAs, 20 DE-lncRNAs and 27 DE-miRNAs. Based on these DE-mRNAs, they were involved in biological processes such as fatty acid beta-oxidation, IRE1-mediated unfolded protein response, and transmembrane transport, and many KEGG pathways like glycine, serine and threonine metabolism, carbon metabolism. Subsequently, lncRNAs *KCTD21-AS1*, *LINC00470* and *SNHG14* were found to be hub nodes in the ceRNA regulatory network. KEGG analysis showed that insulin signaling pathway, glycine, serine and threonine metabolism, pathways in cancer, lysosome, and apoptosis was associated with hypertensive. Finally, insulin signaling pathway was screened to directly associate with HTN and was regulated by mRNAs *PPP1R3C*, *PPKAR2B* and *AKT3*, miRNA has-miR-107, and lncRNAs *SNHG14*, *TUG1*, *ZNF252P-AS1* and *MIR503HG*.

**Conclusions:**

Insulin signaling pathway was directly associated with HTN, and miRNA has-miR-107 and lncRNAs *SNHG14*, *TUG1*, *ZNF252P-AS1* and *MIR503HG* were the biomarkers of HTN. These results would improve our understanding of the occurrence and development of HTN.

**Supplementary Information:**

The online version contains supplementary material available at 10.1186/s12882-020-02142-8.

## Background

Hypertension, which is influenced by genetic and environmental factors, is a risk factor of cardiovascular and cerebrovascular disease. The kidney is not only an important organ causing hypertension, but also the target organ of hypertensive damage [1]. Hypertensive nephropathy (HTN) is a kind of renal injury caused by chronic hypertension [2]. When hypertension lasts for 5–10 years, renal arteriolosclerosis, thickening of tube walls, and narrowing of bureaucratic cavities can occur, which will trigger substantial ischemic injury to the kidney [3]. Although the antihypertensive agent such as cilnidipine and avosentan are commonly used for the treatment of HNT, the effect of clinical treatment is still not ideal [4]. Due to the increasing occurrence and mortality rate of HTN, molecular mechanisms of hypertensive effect on kidney are urgent to be revealed, which is helpful for the early diagnosis and targeted control therapy of HTN.

Previously, a number of biomarkers have been identified to reveal the potential mechanisms of HTN, such as β2-microglobulin (β2-MG), transforming growth factor-β (TGF-β) and periostin [5, 6]. β2-MG, a small globulin, was a good indicator for the diagnosis of early HTN [7]. The high level of β2-MG in serum indicated the damaged filtration function and increased filtration load of glomerular [5]. TGF-β was also reported to be associated with HTN and its high level affected the progression of HTN [8]. Furthermore, periostin, related to proteinuria, plasma creatinine and renal flow, has been identified as the key markers of progression and regression in HTN [6]. Although some protein biomarkers of HTN were identified, there were little researches on gene markers of HTN.

Competing endogenous RNAs (ceRNAs) can regulate gene expression by competing for the binding microRNAs (miRNAs). A ceRNA regulatory network displayed a novel level of long non-coding RNA (lncRNA), microRNA (miRNA) and Mrna [9]. Increasing evidence showed that ceRNAs play important roles in many biological processes, and its imbalance may lead to the initiation and development of human disease [10–12]. By constructed ceRNA regulatory network of gastric cancer, Liu et al. [13] indicated that deleted in lymphocytic leukemia 2 (*DLEU2*) and DX11 antisense RNA 1 (*DDX11-AS1*) were hub lncRNAs in gastric cancer and act as potential ceRNAs to sponge miRNA. LncRNA *Arid2-IR* was one of the most highly up-regulated lncRNAs in mouse model of nonimmune-related kidney diseases and might play a functional role in renal inflammation [14]. In addition, Chen et al. [2] showed that has-miR-335-5p and has-miR-26b-5p, which modulated the majority of differentially expressed RNAs (DERs), were the two most outstanding miRNAs in HTN, by analyzing the miRNA-DERs regulatory network. Although some studies have indicated potential biomarkers associated with HTN, single biomarkers have limitations to explore the mechanism of hypertension on kidney.

In this study, the DERs were screened between hypertensive and normotensive, and then a ceRNA regulatory network was constructed comprised of lncRNA, miRNA and mRNA. Subsequently, Kyoto Encyclopedia of Genes and Genomes (KEGG) analysis of genes existed in the ceRNA regulatory network were performed based on the Gene Set Enrichment Analysis (GSEA). Finally, based on the results of KEGG, a ceRNA regulatory network directly associated with HTN was proposed. These results would improve our understanding of the occurrence and development of HTN.

## Methods

### Data pre-processing and annotation of RNA

We downloaded the dataset numbered GSE28260 from the NCBI Gene Expression Omnibus (GEO, https://www.ncbi.nlm.nih.gov/) on July 26, 2019, which included two cubes: gene expression profile GSE28345 and miRNA expression profile GSE28283 [15]. Each cube contained 8 samples, including 3 samples of normotensive and 5 samples of hypertensive. The renal cortical tissue expression profiles in normotensive and hypertensive were originated from Affymetrix Human Gene 1.0 ST Array and Agilent-021827 Human miRNA Microarray (V3), respectively.

The data of GSE28345 from Affymetrix platform were pre-treated with expression value background correction and normalization by R 3.4.1 oligo package (Version 3.6, http://www.bioconductor.org/packages/release/bioc/html/oligo.html) [16], including conversion of original data format, background correction (MAS method), supplementation of missing value by median method, and data standardization by quantiles method. Afterwards, based on that the one gene can correspond to multiple probes, the data of GSE28283 from Agilent platform was first commented and then calculated the average value of these multiple expression. Subsequently, the data was carried on a logarithm analysis by R 3.4.1 limma package (Version 3.34.0, https://bioconductor.org/packages/release/bioc/html/limma.html) [17].

### Selection of DERs and functional analysis

The lncRNAs, miRNAs and mRNAs detected in expression profile were annotated by HUGO Gene Nomenclature Committee (HGNC) database which including 4409 lncRNAs, 1914 miRNAs and 19,221 protein coding gene (http://www.genenames.org/) [18]. Then the DERs between hypertensive and normotensive groups were screened using R 3.4.1 limma package Version 3.34.0 [17]. False discovery rate (FDR) value < 0.05 and |log_2_ fold change (FC)|>0.5 were set as threshold to identify DERs.

Based on the screened DERs, the pheatmap (Version 1.0.8, https://cran.r-project.org/package=pheatmap) [19] of R 3.4.1 was utilized for bidirectional hierarchical clustering [20] according to the Euclidean distance [21] and was displayed with heat map. Afterwards, Gene ontology (GO) terms and KEGG pathway enrichment analysis of differentially expressed mRNAs (DE-mRNAs) were performed by DAVID (Version 6.8, https://david.ncifcrf.gov/) [22, 23]. The cut-off of the significantly enriched GO terms and pathways was *P* value < 0.05.

### Construction of ceRNA regulatory network and KEGG analysis

We first downloaded the connection pairs of lncRNA-miRNA from DIANA-LncBase (Version 2, http://carolina.imis.athena-innovation.gr/diana_tools/web/index.php) database [24]. Then the differentially expressed regulatory relationship between lncRNA and miRNA was selected according to the miRNA target gene score (miTG-score) > 0.6. The lncRNA-miRNA network was established with the connection pairs of the opposite expression and was visualized by Cytoscape 3.6.1 (http://www.cytoscape.org/) [25]. Afterwards, starBase (Version 2.0, http://starbase.sysu.edu.cn/) database was used to predict the targeted genes regulated by miRNA connected with lncRNA, and miRNA-mRNA network was constructed and visualized. The starBase database provide the predictive information of the targeted genes by five algorithms (targetScan, picTar, RNA22, PITA and miRanda).

Using the above obtained lncRNA-miRNA pairs and mRNA-miRNA pairs, a ceRNA regulatory network was proposed by combining lncRNA-miRNA-mRNA pairs and visualized by Cytoscape 3.6.1. Finally, the KEGG pathway-enrichment analysis of genes included in the ceRNA regulatory network was performed based on the GSEA (http://software.broadinstitute.org/gsea/index.jsp) [26]. GSEA software is used to evaluate the distribution trend of a predefined set of genes in a gene list that is sequenced by phenotypic relevance, thus determining their contribution to the phenotype.

### Establishment of ceRNA regulatory network directly associated with HTN

“Hypertensive renal” as a keyword was searched in Comparative Toxicogenomics Database 2019 update (http://ctd.mdibl.org/) database [27]. The KEGG pathways related to HTN were found and compared with the above KEGG pathways in the ceRNA regulatory network. RNAs associated with HTN were identified and a ceRNA regulatory network directly associated with HTN was constructed with these RNAs.

## Results

### Screening of DERs

**Fig. 1:**
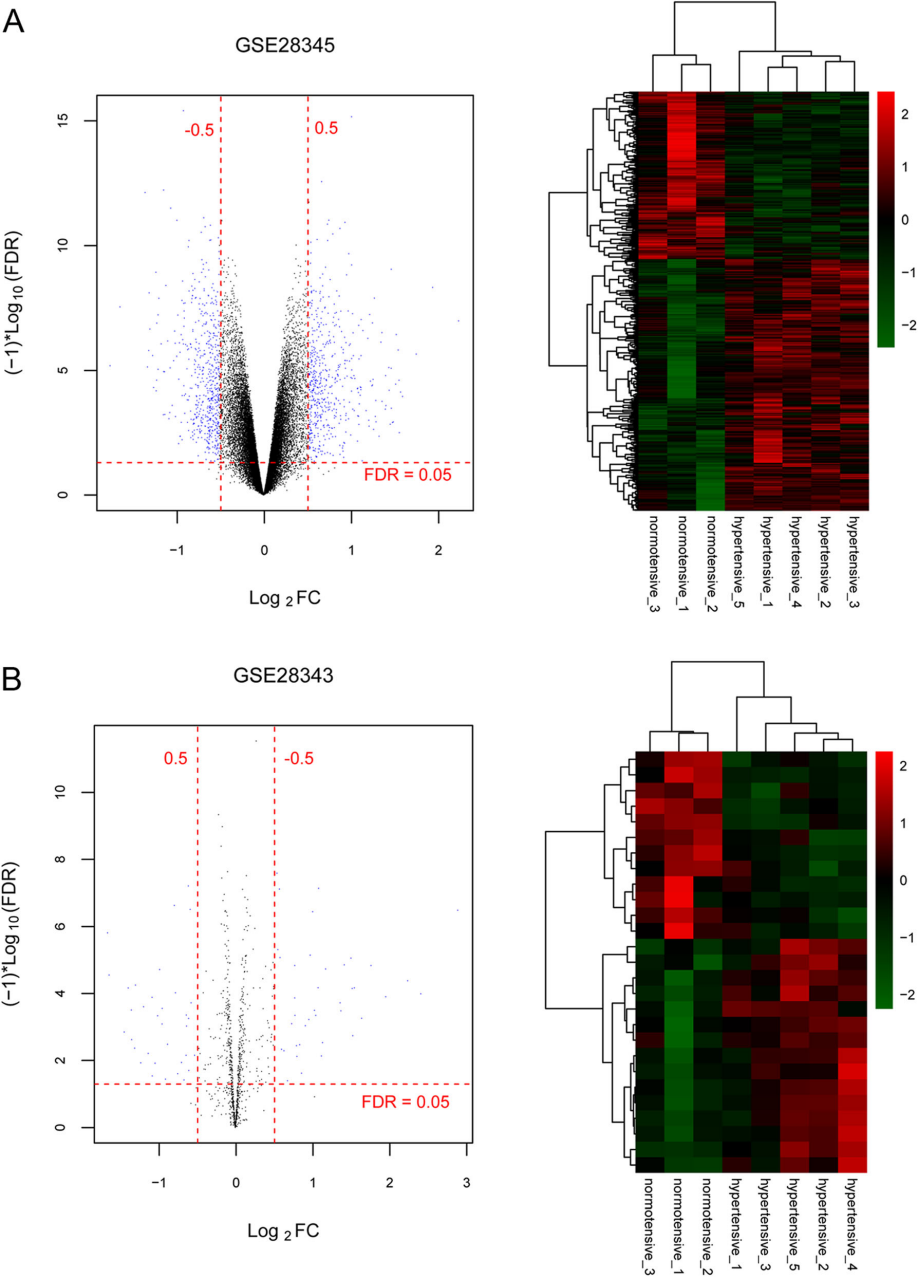
Results of the differentially expressed RNA (DERs). **a** The volcano figure of differentially expressed mRNAs (DE-mRNAs) and differentially expressed lncRNAs (DE-lncRNAs) (left) and differnetially expressed miRNA (DE-miRNAs) (right). Blue dots represented significantly different expression. Red dotted lines represented false discovery rate (FDR) < 0.05. Red vertical dashed lines represented |log2 fold change (FC)|>0.5. **b** Bidirectional hierarchical clustering heatmap of DE-mRNAs, DE-lncRNAs (left) and DE-miRNAs (right)

After annotation by the HGNC database, a total of 459 lncRNAs, 851 miRNAs and 17,962 mRNAs were identified. The DERs between hypertensive and normotensive were screened using limma package, and 947 DERs were filtered based on the thresholds of FDR < 0.05 and |log_2_FC| > 1 (Fig. 1a). These DERs contained 900 DE-mRNAs (352 down-regulated mRNAs and 548 up-regulated mRNAs), 20 DE-lncRNAs (16 down-regulated lncRNAs and 4 up-regulated lncRNAs) and 27 DE-miRNAs (12 down-regulated miRNAs and 15 up-regulated miRNAs). The locations of these DEGs were shown in supplementary Table 1. Based on the expression level of selected DERs, the bidirectional hierarchical clustering was performed. The results showed that the expression value of DERs could well differentiate the hypertensive and normotensive and indicated that the screened DERs were characteristically expressive (Fig. 1b).

### GO terms and KEGG pathways of screened DE-mRNAs

**Fig. 2:**
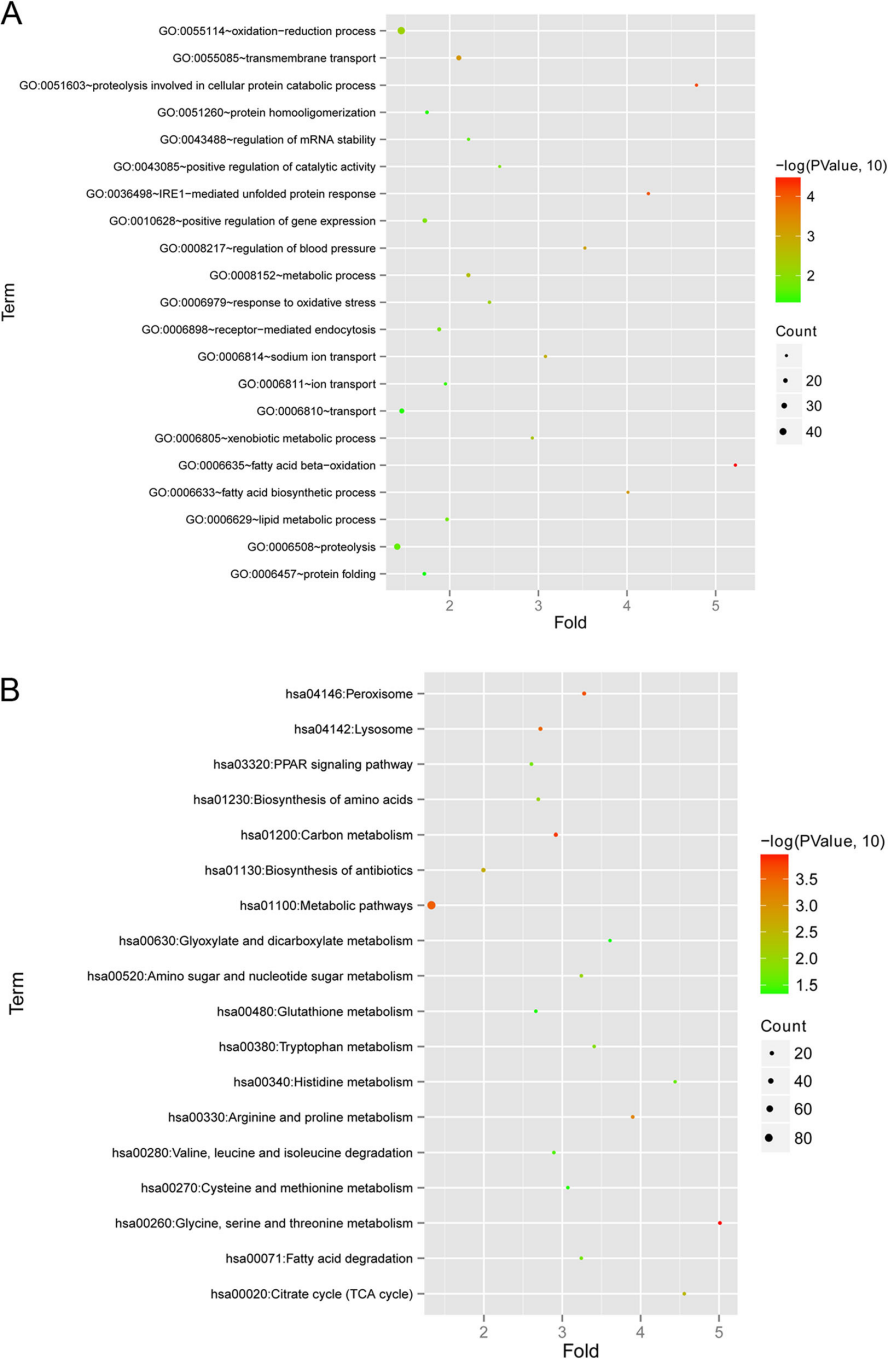
Gene ontological (GO) terms and Kyoto Encyclopedia of Genes and Genomes (KEGG) pathways of DE-mRNAs. **a** The significant GO terms in biological processes. **b** Scatter distribution of KEGG pathways. The size of points represented the number of gene. The colour of points represented the difference of genes

The functional analyses, including GO terms and KEGG pathways, were performed on the above 900 DE-mRNAs, and a total of 21 biological process and 18 KEGG pathways were obtained (Fig. 2). As shown in Fig. 2a, in the involved biological process, the significant enrichment terms of DE-mRNAs were associated with fatty acid beta-oxidation, proteolysis involved in cellular protein catabolic process, IRE1-mediated unfolded protein response, and transmembrane transport. In addition, the DE-mRNAs also played an important role in glycine, serine and threonine metabolism, carbon metabolism, peroxisome, lysosome, metabolic pathways, biosynthesis of antibiotics, arginine and proline metabolism and citrate cycle (TCA cycle) based on the analysis of KEGG (Fig. 2b).

### Construction of ceRNA regulatory network

**Fig. 3:**
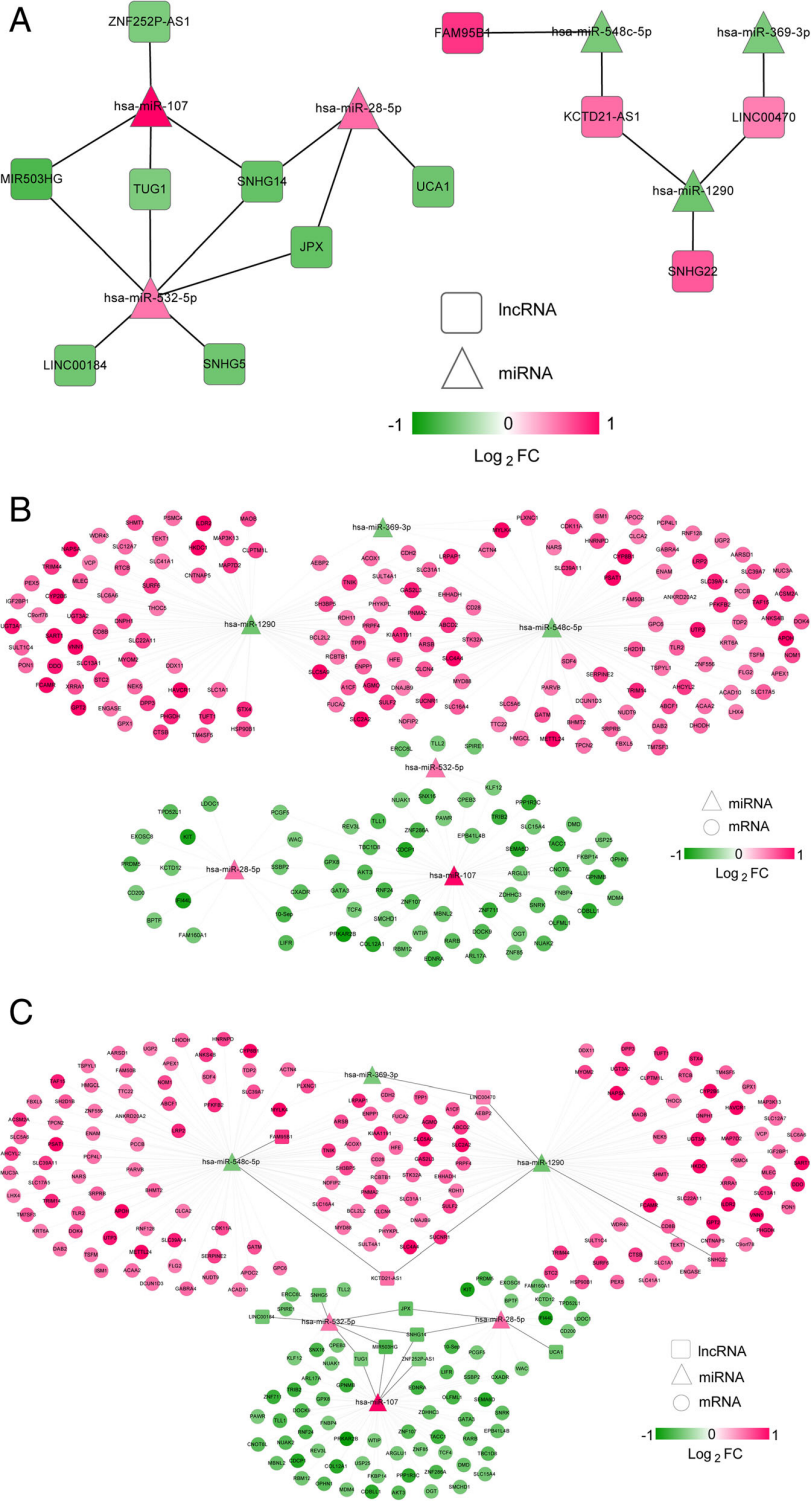
The co-expressed network of (**a**) mRNA-lncRNA, (**b**) mRNA-miRNA and (**c**) mRNA-miRNA-lncRNA (ceRNA regulatory network). The circle represented mRNA, the triangle represented miRNA, and the square represented lncRNA

Before establishing a ceRNA regulatory network, co-expression networks of lncRNA-miRNA and miRNA-mRNA were constructed. The lncRNA-miRNA co-expression network showed that a total of 19 connection pairs were obtained, including 18 nodes, 6 miRNAs (3 down-regulated miRNAs and 3 up-regulated miRNAs) and 12 lncRNAs (8 down-regulated lncRNAs and 4 up-regulated lncRNAs) (Fig. 3a). Afterwards, the results of miRNA-mRNA network displayed that there were 232 nodes in the network and the network contained 6 miRNAs (3 down-regulated miRNAs and 3 up-regulated miRNAs) and 226 mRNAs (68 down-regulated mRNAs and 158 up-regulated mRNAs) (Fig. 3b).

Based on the results of lncRNA-miRNA and miRNA-mRNA, a ceRNA regulatory network consisting of lncRNA-miRNA-mRNA was proposed (Fig. 3c). In the ceRNA regulatory network, 244 nodes were obtained. In addition, 12 lncRNAs (8 down-regulation and 4 up-regulation), 6 miRNAs (3 down-regulation and 3 up-regulation) and 226 mRNAs (68 down-regulation and 158 up-regulation) were identified. Among them, the 8 down-regulated lncRNAs contained *LINC00184*, small nucleolar RNA host gene 5 (*SNHG5*), Jpx transcript Xist activator (*JPX*), urothelial cancer associated 1 (*UCA1*), small nucleolar RNA host gene 14 (*SNHG14*), MIR503 host gene (*MIR503HG*), taurine up-regulated 1 (*TUG1*), ZNF252P antisense RNA 1 (*ZNF252P-AS1*), and the 4 up-regulated lncRNAs included family with sequence similarity 95 member B1 (*FAM95B1*), *LINC00470*, KCTD21 antisense RNA 1 (*KCTD21-AS1*), small nucleolar RNA host gene 22 (*SNHG22*). The 6 miRNAs included hsa-miR-548c-5p, hsa-miR-369-3p, has-miR-1290, has-miR-532-5p, has-miR-28-5p and has-miR-107. Furthermore, in this ceRNA regulatory network, lncRNAs *KCTD21-AS1*, *LINC00470* and *SNHG14* were hub nodes, which targeted more miRNAs and mRNAs.

### KEGG enrichment-pathway analysis of mRNA in the ceRNA regulatory network based on GSEA

After the establishment of ceRNA regulatory network, the KEGG pathways were analyzed by GSEA. In the GSEA results, there are three key statistical values, including enrichment score (ES), normalized enrichment score (NES) and nominal *P* value. Based on the above three key statistical values, five significant KEGG signaling pathways (insulin signaling pathway, glycine, serine and threonine metabolism, pathways in cancer, lysosome, and apoptosis) were identified with the cut-off of *P* < 0.05 (Table 1). Obviously, mRNA protein kinase cAMP-dependent type II regulatory subunit beta (*PRKAR2B*) and AKT serine/threonine kinase 3 (*AKT3*) were both related to the pathways of insulin signaling pathway and apoptosis. *AKT3*, KIT proto-oncogene, receptor tyrosine kinase (*KIT*) and retinoic acid receptor beta (*RARB*) were also involved in pathways in cancer. Furthermore, mRNA protein phosphatase 1 regulatory subunit 3C (*PPP1R3C*) was also essential for insulin signaling pathway.

**Table 1:** KEGG enrichment-pathway analysis of mRNA in the ceRNA regulatory network based on GSEA

Name	Size	ES	NES	NOM *P*-value	Gene
KEGG _ insulin _ signaling _ pathway	3	−0.8969	−1.9100	1.193E-03	PPP1R3C, PRKAR2B, AKT3
KEGG _ glycine _ serine _ and _ threonine _ metabolism	5	0.7330	1.6968	6.270E-03	MAOB, SHMT1, GATM, PSAT1, PHGDH
KEGG _ pathways _ in _ cancer	3	−0.6399	−1.5624	2.632E-02	AKT3, KIT, RARB
KEGG _ lysosome	5	0.6561	1.5132	3.030E-02	NAPSA, SLC17A5, TPP1, ARSB, CTSB
KEGG _ apoptosis	2	−0.6784	−1.4150	4.599E-02	PRKAR2B, AKT3

### Establishment of ceRNA regulatory network directly associated with HTN

**Fig. 4:**
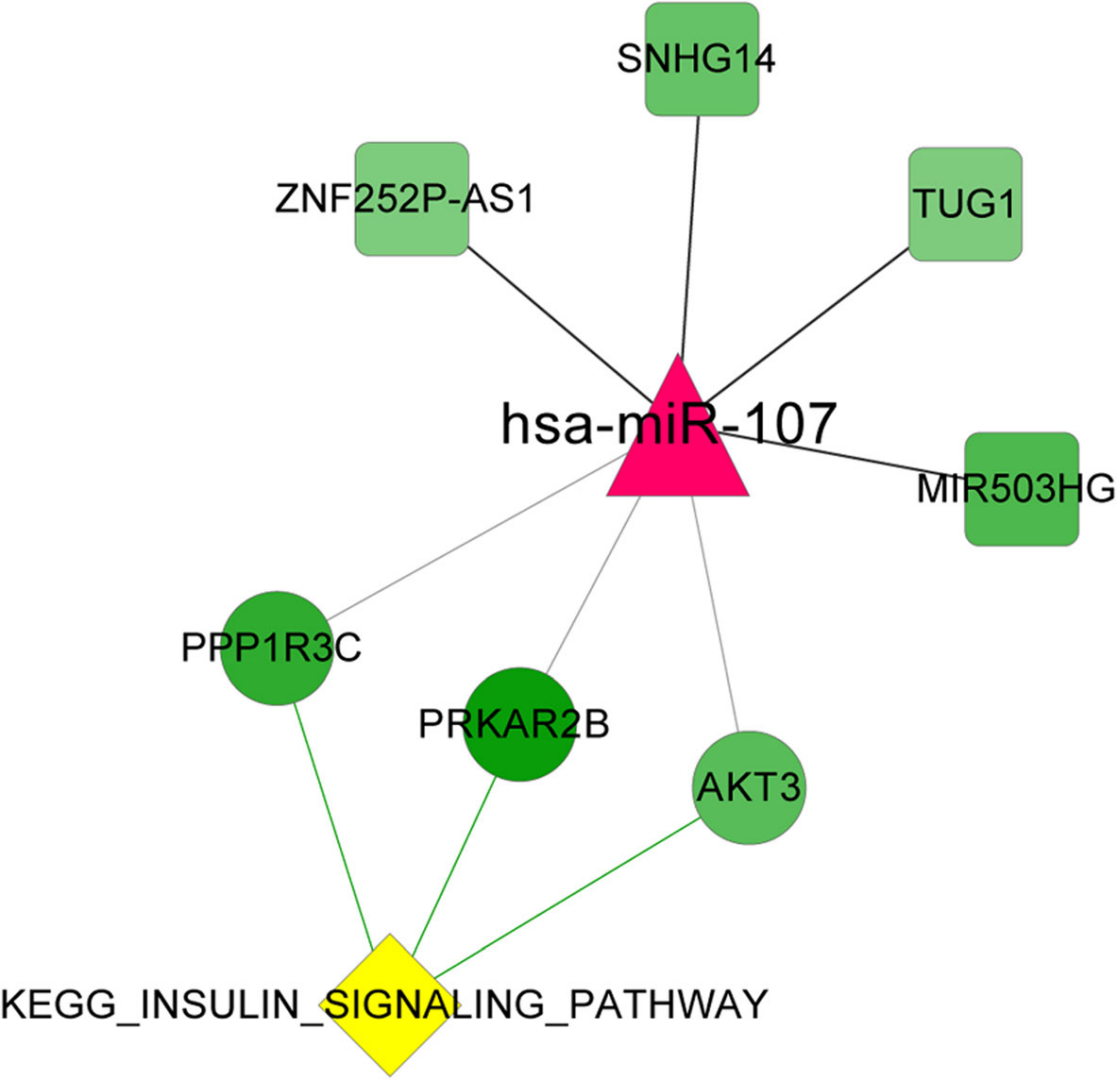
The ceRNA network directly associated with hypertensive nephropathy. The yellow prismatic represented KEGG pathway. The circle represented mRNA, the triangle represented miRNA, and the green square represented lncRNA

In order to better understand the potential biomarkers directly associated with HTN, the KEGG pathways related to HTN were obtained according to the CTD database. After compared with the above KEGG pathways of mRNAs, one pathway directly associated with HTN (insulin signaling pathway) was screened and contained three important mRNAs (*PPP1R3C*, *PPKAR2B* and *AKT3*). Based on the three genes, a ceRNA regulatory network directly associated with HTN was established. As shown in Fig. 4, insulin signaling pathway was regulated by mRNAs *PPP1R3C*, *PPKAR2B* and *AKT3*, miRNA has-miR-107, and lncRNAs *SNHG14*, *TUG1*, *ZNF252P-AS1* and *MIR503HG*.

## Discussion

Recently, HTN is an increasingly common kidney disease in the patients with hypertension, which seriously endangers people’s health. However, the possible mechanism of hypertension affecting HTN remains unclear. In this study, we identified 947 DERs, including 900 DE-mRNAs, 20 DE-lncRNAs and 27 DE-miRNAs. Based on the selected DE-mRNAs, it was found that these mRNAs were involved in biological processes of fatty acid beta-oxidation, proteolysis involved in cellular protein catabolic process, IRE1-mediated unfolded protein response, and transmembrane transport, and many KEGG pathways like glycine, serine and threonine metabolism, carbon metabolism, metabolic pathways, biosynthesis of antibiotics, and citrate cycle (TCA cycle). Afterwards, a ceRNA regulatory network was established with 12 lncRNAs, 6 miRNAs and 226 mRNAs. It is clear that lncRNAs *KCTD21-AS1*, *LINC00470* and *SNHG14* were hub nodes in the ceRNA regulatory network, which targeted more miRNAs and mRNAs. Then KEGG analysis showed that mRNAs included in this network played important roles in insulin signaling pathway, glycine, serine and threonine metabolism, pathways in cancer, lysosome, and apoptosis. Finally, a ceRNA regulatory network directly associated with HTN was proposed. Insulin signaling pathway was screened to directly associate with HTN and was modulated by mRNAs *PPP1R3C*, *PPKAR2B* and *AKT3*, miRNA has-miR-107, and lncRNAs *SNHG14*, *TUG1*, *ZNF252P-AS1* and *MIR503HG*.

At the beginning, 947 DERs were screened out and were found to play roles in many biological processes and signaling pathways. In this study, the screened DERs between hypertensive and normotensive were significantly associated with fatty acid beta-oxidation, IRE1-mediated unfolded protein response, and transmembrane transport. It’s reported that fatty-acid is an effective energy source that lead to the generation of ATP, and involved in the lipid metabolism of cancer cells, such as breast cancer cells [28, 29]. Plate et al. [30] indicated that the activation of IRE1-mediated unfolded protein response could influence endoplasmic reticulum quality control, and secretory proteostasis for destabilized, disease-relevant proteins. Furthermore, the damage of transmembrane transport would result in the imbalance of salt and water, and abnormality of blood pressure, which finally caused renal disease [31]. Therefore, fatty acid beta-oxidation, IRE1-mediated unfolded protein response, and transmembrane transport might play important roles in HTN. In addition, in our study, these DERs were also participated in many KEGG pathways like glycine, serine and threonine metabolism, carbon metabolism, biosynthesis of antibiotics, and citrate cycle (TCA cycle). Recent studies have shown that glycine, serine and threonine metabolism, and carbon metabolism affected the antioxidant ability of cells and their hyperactivation would drive the occurrence of cancer [32, 33]. However, the pathways of biosynthesis of antibiotics, and citrate cycle (TCA cycle) were not clear in HTN.

To understand the relationship between target genes and functions, a ceRNA regulatory network was established composing with 12 lncRNAs, 6 miRNAs and 226 mRNAs. In this network, lncRNAs *KCTD21-AS1*, *LINC00470* and *SNHG14* were found to be hub nodes. *KCTD21-AS1*, which promoted protein degradation and reduced cellular signaling, was associated with many diseases, including breast cancer [34] and obesity [35]. A study by Liu et al. indicated that *LINC00470* was up-regulated in glioblastoma cell and its high level expression was an unfavorable prognosis marker for astrocytoma patients [36]. Moreover, *SNHG14*, as a key lncRNA, facilitated the migration and invasion of clear cell renal cell carcinoma via sponging miR-203 and accelerate Neural Wiskott-Aldrich syndrome protein [37]. Therefore, we supposed that lncRNAs *KCTD21-AS1*, *LINC00470* and *SNHG14* may be related to HTN.

In the end, a ceRNA regulatory network directly associated with HTN was proposed to find the biomarkers directly related to HTN. Our study showed that insulin signaling pathway was screened to directly associate with HTN. Insulin signaling pathway was reported to be damaged in the glomerulus by high glucose and promote apoptotic environment [38]. Therefore, insulin signaling pathway is possible to be a target for the prevention and treatment of HTN. In addition, based on the ceRNA regulatory network directly associated with HTN, insulin signaling pathway was modulated by mRNAs *PPP1R3C*, *PPKAR2B* and *AKT3*, miRNA hsa-miR-107, and lncRNAs *SNHG14*, *TUG1*, *ZNF252P-AS1* and *MIR503HG*. MiRNA hsa-miR-107, as the hub in this network, was reported to be overexpressed in gastric carcinoma and promote tumor growth and survival [39]. Many researchers have shown that *PPP1R3C*, *PPKAR2B* and *AKT3* may play important roles in cervical cancer [40], cardiovascular events [41] and prostate cancer [42]. In addition, lncRNAs *TUG1*, an important regulator of cancers, could facilitate proliferation and suppressed apoptosis by regulating miR-132-3p in osteosarcoma cells [43]. *MIR503HG* could inhibit migration and invasion of cells via miR-103/OLFM4 axis in triple negative breast cancer. However, the studies with regard to *ZNF252P-AS1* were few. Therefore, combined with our results, lncRNAs *SNHG14*, *TUG1*, *ZNF252P-AS1* and *MIR503HG* were the biomarkers of HTN and HTN may be affected by insulin signaling pathway regulated by has-miR-107.

However, there were some limitations of our study. Firstly, the sample size is not big enough and the research was lack of validation of experiment. Furthermore, studies of these biomarkers need to be further investigated on the clinical practice, and one signaling pathway obtained by bioinformatics analysis needs to be verified in an animal model or human biopsies from patients.

## Conclusion

We identified 947 DERs, including 900 DE-mRNAs, 20 DE-lncRNAs and 27 DE-miRNAs. These DE-mRNAs were involved in the fatty-acid beta-oxidation, IRE1-mediated unfolded protein response, and transmembrane transport, and KEGG pathways like glycine, serine and threonine metabolism. Based on the ceRNA regulatory network, *KCTD21-AS1*, *LINC00470* and *SNHG14* were hub nodes, which targeted more miRNAs and mRNAs. Finally, the ceRNA regulatory network directly associated with HTN revealed that insulin signaling pathway was directly related to HTN and found the pathway was regulated by 3 mRNAs (*PPP1R3C*, *PPKAR2B* and *AKT3*), 1 miRNA (has-miR-107) and 3 lncRNAs (*SNHG14*, *TUG1*, *ZNF252P-AS1* and *MIR503HG*). These results indicated that *SNHG14*, *TUG1*, *ZNF252P-AS1* and *MIR503HG* regulated by has-miR-107 may be the biomarkers closely associated with HTN and would improve our understanding of the occurrence and development of HTN.

## Supplementary Information

**Additional file 1.**

## Data Availability

The datasets used and/or analysed during the current study are available from the corresponding author on reasonable request.

## Abbreviations

HTN: Hypertensive nephropathy
DER: Differentially expressed RNA
GSEA: Gene set enrichment analysis
GO: Gene ontology
KEGG: Kyoto encyclopedia of genes and genomes
ceRNA: Competing endogenous RNA
